# A Self-Adaptive and Self-Sufficient Energy Harvesting System

**DOI:** 10.3390/s20092519

**Published:** 2020-04-29

**Authors:** Mario Mösch, Gerhard Fischerauer, Daniel Hoffmann

**Affiliations:** 1 Chair of Measurement and Control Systems, Center of Energy Technology (ZET), Universität Bayreuth, Universitätsstraße 30, D-95447 Bayreuth, Germany; mrt@uni-bayreuth.de; 2 Hahn-Schickard, Wilhelm-Schickard-Str.10, D-78052 Villingen-Schwenningen, Germany; daniel.hoffmann@hahn-schickard.de

**Keywords:** vibration energy harvesting, self-adaptive, self-sufficient, electromagnetic, frequency measurement, microgenerator

## Abstract

Self-adaptive vibration energy harvesters convert the kinetic energy from vibration sources into electrical energy and continuously adapt their resonance frequency to the vibration frequency. Only when the two frequencies match can the system harvest energy efficiently. The harvesting of vibration sources with a time-variant frequency therefore requires self-adaptive vibration harvesting systems without human intervention. This work presents a self-adaptive energy harvesting system that works completely self-sufficiently. Using magnetic forces, the axial load on a bending beam is changed and thus the resonance frequency is set. The system achieves a relative tuning range of 23% at a center frequency of 36.4 Hz. Within this range, the resonance frequency of the harvester can be set continuously and precisely. With a novel optimized method for frequency measurement and with customized electronics, the system only needs 22 µW to monitor the external vibration frequency and is therefore also suitable for environments with low vibration amplitudes. The system was verified on a vibrational test bench and can easily be tailored to a specific vibration source.

## 1. Introduction

Vibration energy harvesters are an alternative to batteries for operating wireless sensor networks (WSN). They are advantageous in that they provide energy for longer periods of time than a battery in places with sufficient vibration and are more sustainable [1,2]. Examples of WSNs powered by kinetic energy harvesters are wearable devices [3,4,5], wild animal tracking devices [6], condition monitoring devices [7], networks for the early detection of natural disasters [8,9], and local monitoring devices [10]. A sensor node should run self-sufficiently after the first installation, so there is no need for user intervention for reasons of cost, comfort and accessibility.

Vibration harvesters perform best when their resonance frequency matches their vibration frequency. When the two frequencies differ, the harvested power decreases sharply [11]. Then, an electrical load, e.g., a sensor node, can no longer be supplied with sufficient energy. In environments with a time-varying vibration frequency, e.g., a gearbox [12] or household devices like washing machines or kitchen hoods [13], a harvester design that ensures the highest possible harvested power is therefore required. There are two methods by which this can be achieved. The first way is by broadening the resonance curve of the harvester, e.g., by using nonlinear energy harvesters [14] or frequency up-conversion [15,16]. We used the second approach, by which a harvester continuously adjusts its resonance frequency to match the vibration frequency. These systems are called self-adaptive energy harvesters.

The resonance frequency $f_{r}$ of a mechanical mass-spring-damper system is determined by the effective mass *m* and the spring stiffness *k* of the harvester:

(1) $$f_{r}\approx f_{n}=1/(2\pi )\cdot \sqrt{k/m}.$$

The resonance frequency $f_{r}$ can be approximated to the natural frequency $f_{n}$ for a high Q-factor [17], which is the case for our vibration harvester. The adaptation techniques therefore aim to change either the mass or the stiffness. Various methods have been developed and investigated in the past [11,18]. Heating the structure changes the Young’s modulus and therefore the stiffness [19,20], but consumes too much power. Manipulating the clamping length of a cantilever structure also changes the stiffness, but is more suitable for a manual adaptation [21]. The same applies to a change in the center of gravity, e.g., by moving a screw at the free end of a cantilever [22], or a change in the proof mass geometry [23]. The resonance frequency $f_{r}$ can also be changed via an additional spring stiffness $k_{a}$ [11]:

(2) $$f_{r}\approx 1/(2\pi )\cdot \sqrt{(k+k_{a})/m}.$$

Challa et al. built a cantilever harvester with magnets on the free end [24]. Fixed magnets above and below this magnet provide additional spring stiffness $k_{a}$ due to their interacting magnetic forces. This additional stiffness can also be realized via electrostatic [25] or piezoelectric forces [26]. Applying an axial load to a beam structure also changes its resonance frequency. For a bending beam clamped on one side, the relative change in the resonance frequency is [11]:

(3) $$\frac{f_{r}+\Delta f_{r}}{f_{r}}=\sqrt{1+\frac{F_{a}}{F_{E}}}=\sqrt{1+F_{a}\frac{48\ell^{2}}{\pi^{2}Ewd^{3}}},$$

where the axial and Euler loads are $F_{a}$ and $F_{E}$, the clamping length ℓ, the Young’s modulus *E*, and the beam width and thickness *w* and *d*. The axial force $F_{a}$ is positive for tensile and negative for compressive loads. A tensile load therefore increases, and a compressive load reduces, the resonance frequency $f_{r}$. However, the force $F_{a}$ must not exceed the Euler buckling load $F_{E}$, otherwise the beam will buckle and this will lead to nonlinear behavior [27]. Axial forces can easily be generated with magnetic forces [28]. If a magnet is attached to the end of a bending beam and a second magnet moves along the beam axis towards the first magnet, the axial force increases. The acting force is respectively compressive and tensile when like and unlike magnetic poles point towards each other. Axial loads can also be produced via piezoelectric forces [29]. Among these examples, the axial load method and the additional spring stiffness method are particularly suitable for a self-adaptive mode of operation, since a large adaption frequency bandwidth and self-sufficient operation are possible. Eichhorn et al. further developed their adaptive harvester [29] to a self-adaptive system [30]. The system is powered by its own harvested energy and runs self-sufficiently, but can only adapt to lower frequencies. In addition, the piezoelectric tuning mechanism regularly requires energy to maintain an actuator voltage and thus a state of adaptation. This semi-active adjustment is inferior to a passive adjustment, where the system only needs energy for the transition to a new resonance frequency, but not to hold the frequency. The term “passive” does in this context not mean an adapting system totally without the need of adjustment energy [31]. Environments with a low vibration do not offer much energy to harvest, so it is vital to save as much energy as possible. 

A completely self-sufficient system with passive adaptation has not been described in the literature to the authors’ knowledge. The development and characterization of such a system is the topic of this work. The adaptation is carried out by a rotatable diametrically magnetized tuning magnet, which, depending on the angle of rotation, applies a specific axial load to a bending beam harvester and thus changes the resonance frequency. The rotation is performed by a stepper motor. The design of the structure is inspired by the self-adaptive system created by Hoffmann et al. [32], which represents an intermediate step to an autonomous system. Our system additionally includes a self-sufficient adaptation algorithm and an energy-efficient vibration frequency measurement. This allows for an energy-efficient and autonomous operation, with a power consumption of only 22 µW (without counting the adaptation energy). The setup is a prototype to test and optimize the function of the individual components and their interaction under defined conditions before the field use of the system. During the tests on the vibrational test bench, the frequency of the harmonic vibration is changed at certain time intervals; this simulates the typical situation of a time-variant vibration source. Some examples include, as mentioned above, gear boxes [12], household devices [13] or, more generally, machines that involve components rotating at variable speeds [30]. An extensive characterization of these sources showed that the vibrations associated with them consist of clearly separated harmonics that change with the operating settings. They do not exhibit a broadband behavior such as white noise and therefore call for resonant (narrowband) energy harvesters. Our general structure can be individually tailored to the source by changing the beam geometry.

The paper is organized as follows. Section 2 describes the design of the energy harvesting system and its individual parts. In Section 3, we report on experiments using a vibrational test bench and discuss their results as a means of characterizing the novel system. Section 4 provides a summary.

## 2. System Setup

### 2.1. Overview

**Figure 1:**
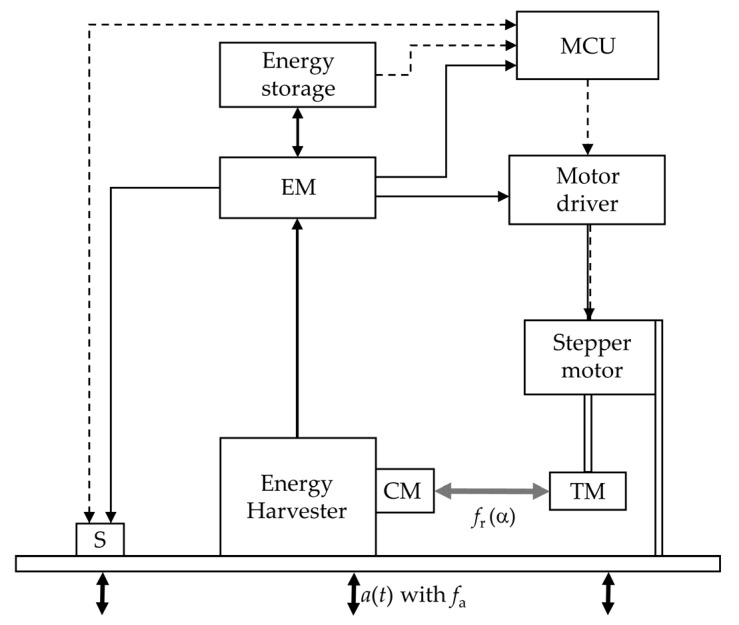
Sketch of the self-adaptive energy harvester on the test bench. Dashed lines indicate data exchange, solid lines indicate energy flow. Coupling magnet (CM), tuning magnet (TM), acceleration sensor (S), energy management (EM), microcontroller (MCU).

The complete system, with its individual components, is outlined in Figure 1. The energy harvester (EH) converts the energy associated with the vibration, represented by the acceleration a(t) with the frequency $f_{a}$, into electrical energy via electromagnetic coupling. The harvester design is based on the known cantilever structure [33,34], whereby the beam is clamped on one side and permanent magnets are attached to the free end, which oscillate relative to a copper coil. On the free beam end, there is a coupling magnet (CM), which does not play a role in energy conversion, but transmits the force that is exchanged with the tuning magnet (TM) as an axial load on the bending beam. The tuning magnet is diametrically magnetized and is attached to the shaft of the stepper motor.

The energy management system (EM) stores the harvested energy in an energy storage and provides a constant voltage of 3.0 V for electrical loads. The circuit is described in more detail in Hoffmann et al. [32]. In this work, the energy storage system is a supercapacitor with a capacitance of C=0.25 F.

The microcontroller (MCU) analyzes the measured acceleration data and executes the adaptation algorithm. When choosing the MCU, attention must be paid to its low standby consumption and its energy-efficient processing of the code. An STM32L452 from STMicroelectronics was used in this work. The MCU runs at a clock rate of 16 MHz.

The acceleration sensor (S) measures the acceleration and transmits the data to the MCU. For this purpose, it must run energy-efficiently and have a sufficiently high measuring rate. The energy harvester is designed for vibration frequencies of up to 40 Hz, so a measuring rate of more than 80 Hz is required [35] (pp. 1–15). We chose a measuring rate which exceeds the vibration frequency by a factor of between five and 10, which also decreases the leakage effect in the spectrum [35] (pp. 1–23). The ADXL362 sensor from Analog Devices measures accelerations with a sampling rate of up to 400 Hz with very low consumption. The measurement options are set via registers before measurements start. Communication with the MCU occurs via the serial peripheral interface (SPI) bus. The sensor stores the measurement data in a first-in, first-out (FIFO) memory, which the MCU reads via direct memory access (DMA).

The motor driver is the interface between the MCU and the stepper motor. The driver converts a desired motor movement into an actual movement of the stepper motor using pulse width-modulated (PWM) control signals. The driver must be able to move the motor with a supply voltage of 3.0 V and have a low standby consumption. In this work, owing to the high compatibility with the MCU, the stepper motor driver STSPIN220 from STMicroelectronics was used on the development board X-NUCLEO-IHM06A1. Communication with the MCU occurs via the SPI bus.

The motor is required to have as high a residual torque as possible to avoid step loss during the adaptation or the holding time afterwards. With unnoticed step losses, the tuning magnet would be positioned at the wrong rotation angles, which would lead to the wrong resonance frequencies and a reduced harvested power. The used stepper motor complies with the NEMA (National Electrical Manufacturers Association) 17 standard and has a step angle of 1.8°. The motor driver and the motor are powered by the system’s energy storage.

### 2.2. Adaptivity and Bandwidth

**Figure 2:**
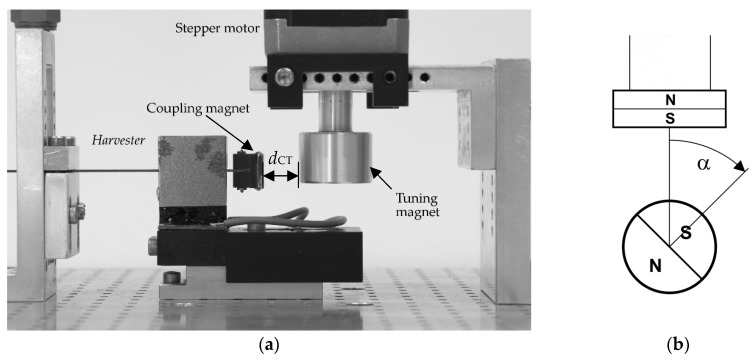
Self-adaptive energy harvesting system with electromagnetic tuning. (**a**) Harvester prototype on the test bench. *d*_CT_ is the magnet gap. (**b**) The angle α is defined by the position of the south (S) and north (N) poles between the coupling magnet (top) and tuning magnet (bottom).

The energy harvester converts the vibration energy into electrical energy via electromagnetic coupling and, as mentioned, is based on the bending beam design. The authors have already published a smaller but similar model without a coupling magnet at the free end [36]. The beam is made of copper and is 0.7-mm thick and 10-mm wide. The free bending length of 23 mm lies between the clamping and the induction magnets (see Figure 2a). The induction magnets have a 6-mm gap in which the copper coil sits connected to the ground (the beam also has a gap here). The coupling magnet is mounted on the 6-mm long free end of the beam and is glued to a plastic holder bolted to the beam. The beam has an overall length of 53 mm from the clamping point to the coupling magnet.

The induction magnets and the coupling magnet have the dimensions 20 × 10 × 2 mm. The magnetic flux is guided by iron legs measuring 20.7 × 20 × 1 mm. The tuning magnet measures 5 mm in height and 18 mm in diameter and is attached to the shaft of the stepper motor with an aluminum holder. All magnets are made of NdFeB. The cylindrical air copper coil has an inside and outside diameter of 2.9 and 20 mm and a thickness of 3 mm. A wire diameter of 50 µm results in 5690 coil turns and a coil resistance of 1.77 kΩ (measured with a multimeter).

The resonance frequency of the beam depends on the angle α and the magnet distance $d_{\mathrm{CT}}$ (see Figure 2). The adaptivity range $\Delta f_{\mathrm{AB}}$ is defined by the resonance frequencies $f_{r}$ that the harvester can attain. The lower and upper limits of the adaptation range correspond to the angles for maximum axial pressure and tension on the beam (α=0° and α=180°, see Equation (3)). With a neutral position α=90° and the middle frequency $f_{r,M}$, there is no axial load. The smaller the magnet spacing $d_{\mathrm{CT}}$, the greater the load on the beam and the greater $\Delta f_{\mathrm{AB}}$. If the distance is too short, either the residual torque of the motor or the Euler buckling load $F_{E}$ (Equation (3)) are exceeded; in the first case there is a step loss, in the second there is unwanted nonlinear behavior.

The energy harvesting test bench consisted of a waveform generator connected to a permanent magnet shaker (B&K LDS V406, Nærum, Denmark), an accelerometer (B&K 4534-B-001) for measuring the vibration strength, and a voltage measurement card (NI PXIe-6341, Austin, TX, USA). The measurement card recorded the load or open circuit voltages and the acceleration sensor output voltage. The whole bench was controlled by a personal computer, so the user could vary the frequency and the acceleration amplitude. The step width of the frequency sweep was set to 50 mHz. The same test bench was previously used in [36].

**Figure 3:**
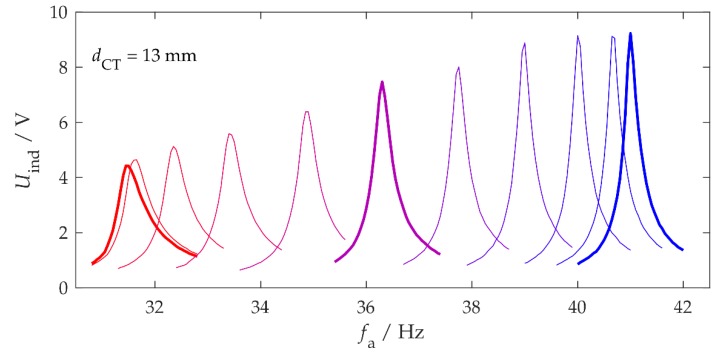
Resonance curves of the open circuit voltage for different rotation angles α between α = 0° (red) and α = 180° (blue). The magnet distance was *d*_CT_ = 13 mm.

The adaptivity behavior was tested on the test bench for different rotation angles α and magnet distances $d_{\mathrm{CT}}$, with an acceleration amplitude of $A=0.5\;{m/s}^{2}$. $d_{\mathrm{CT}}=13\;\mathrm{mm}$ was the smallest distance at which the motor could keep the magnet at rest. Figure 3 shows the resonance curves of the induction voltage for different angles, which are color-coded; red corresponds to repulsive magnetic forces (α=0°) and blue to attractive forces (α=180°). α was changed in constant steps of 18° by the MCU.

The resonance curves shown differ in width and height. Stronger axial tensile loads of the bending beam reduced the mechanical damping ratio $\zeta_{m}$ and increased the quality factor $Q_{m}=1/(2\zeta_{m})$. Conversely, mechanical forces that compressed the structure to change the resonance frequency increased the mechanical damping [24,29]. Here, $Q_{m}$ was between 60 and 170. With the connected energy management, which acted as an electrical load and accordingly corresponded to additional electrical damping, the total Q-factor at all frequencies was approximately 40.

For geometric reasons, $f_{r}(\alpha )$ can be described using a trigonometric function:

(4) $$f_{r}(\alpha )=f_{r,M}+\frac{\Delta f_{\mathrm{AB}}}{2}(1-\cos\alpha ).$$

**Figure 4:**
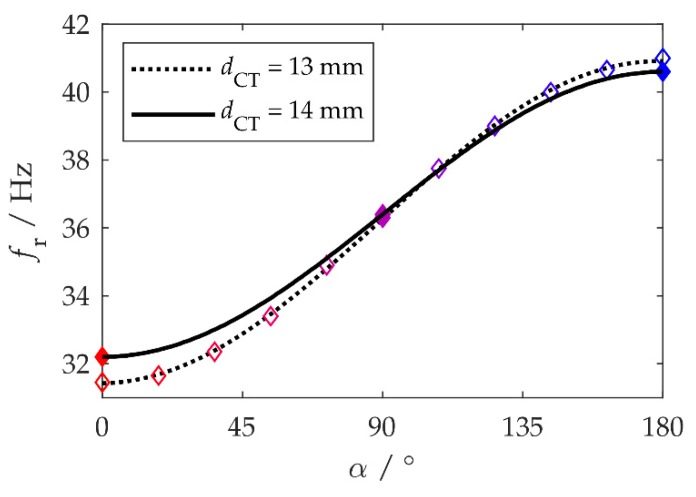
Relationship between the magnet angle α and the resonance frequency *f*_r_. The unfilled and filled diamonds, respectively, represent the measured resonance frequencies *f*_r_ for *d*_CT_ = 13 mm (see Figure 3) and *d*_CT_ = 14 mm. The solid and dotted lines are the results of fitting the trigonometric function (4) to the measurement data.

Fitting this trigonometric function to the measured resonance frequencies led to convincing coefficients of determination ($R^{2}\approx 1$; see Figure 4, unfilled diamonds and dotted line). It therefore suffices to measure a few resonance frequencies (say, at α=0°, α=90°, and α=180°) and then determine the coefficients $f_{r,M}$ and $\Delta f_{\mathrm{AB}}$ of the adaption (Equation (4)) by fitting.

Extensive testing of the system revealed that at a distance of $d_{\mathrm{CT}}=13\;\mathrm{mm}$, the angle α was maintained during hold phases, but sometimes step losses occurred during rotations Δα>0. Owing to the attractive magnetic forces during this movement, the motor occasionally rotated too far. The distance was therefore increased to $d_{\mathrm{CT}}=14\;\mathrm{mm}$ in order to lower the magnetic forces. This led to an adaptation bandwidth of $\Delta f_{\mathrm{AB}}=8.4\;\mathrm{Hz}$ and a center frequency *f*_r,M_ = 36.4 Hz (Figure 4, filled diamonds and solid line). The magnetic forces now no longer caused step losses, so all further tests were carried out with this setting.

### 2.3. Stepper Motor

In the operation of the stepper motor, the main goal was to avoid step losses. At the same time, the adaptation must be as energy efficient as possible, so the motor should use as little energy as possible per angle shift Δα. Parameters such as the acceleration rate, the braking rate, and the speed of rotation were determined by the motor driver settings. At $d_{\mathrm{CT}}=14\;\mathrm{mm}$, and with appropriate motor settings, the angle shift-dependent adaptation energy $W_{T}$ was found to be:

(5) $$W_{T}(\Delta \alpha )=22\;\mathrm{mJ}+1.2\;\mathrm{mJ}/{}^{\circ}\cdot |\Delta \alpha |.$$

This equation resulted from the linear fit of the experimental observations with over 20 various angle changes in the direction of both repulsive and attractive magnetic forces (coefficient of determination $R^{2}\approx 0.95$). The motor driver and the motor were connected to a supercapacitor and the adaptation energy was calculated by the drop in the capacitor voltage. The offset of 22 mJ is due to the braking and acceleration phases, which occurred during each adaptation. One should assume that the rotation towards repulsive forces requires more energy, but this difference, although observable, was negligible. Rotations with |Δα|<8° led to repeated step losses in the experiment and were avoided for this reason.

### 2.4. Vibration Frequency Measurement

The sensor measures the acceleration a(t) with a sampling rate of 400 Hz and transmits the values to the MCU via the SPI bus. The MCU performs fast Fourier transform (FFT) with the digital signal processing (DSP) library from STMicroelectronics [37]. The precise measurement of the dominant vibration frequency $f_{a}$ is crucial for the operation of a self-adaptive system. Owing to the narrow resonance curves of the harvester (see Figure 3), $f_{a}$ needs to be determined with an accuracy of 0.1 Hz with as little energy as possible.

In the worst case, $f_{a}$ is exactly between two points in the discrete frequency spectrum, so that a frequency resolution of $\Delta f_{S}=0.2\;\mathrm{Hz}$ is necessary, which corresponds to a measurement duration of *T* = 5 s. With the 400 Hz sampling rate, the transmission, storage, and evaluation of 2000 values is necessary (to be more precise, 2^11^ = 2048 values are required because of the limitation of the library function to powers of two). In the case of clearly defined vibration frequencies, the measurement duration can be reduced. A total of 1024 measured values are recorded in T=2.56 s, so that the sensor only runs half of the time compared to the longer measurement. After zero-padding the measurement data to 2048 measurement points [35] (p. 31), the spectrum provides a graphic resolution of $\Delta f_{S}=0.2\;\mathrm{Hz}$. However, an FFT with 2048 measuring points is still required.

**Figure 5:**
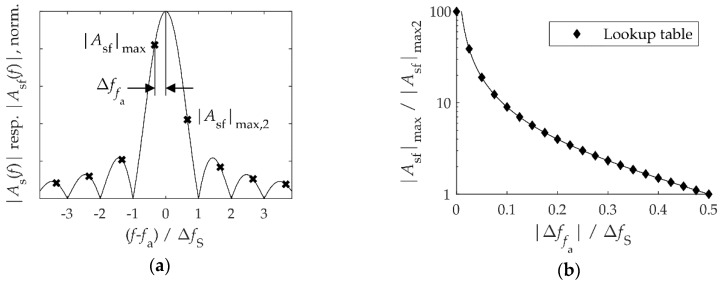
Evaluation of the vibration frequency *f*_a_ from a discrete spectrum involving the leakage effect: (**a**) The amplitude spectrum |*A*_s_(*f*)| (solid line) and discrete spectrum |*A*_sf_(*f*)| (crosses) of a harmonic signal with the frequency *f*_a_ measured over a time *T*, which is not an integer multiple of 1/*f*_a_. (**b**) The ratio of the two largest values in the discrete spectrum according to Equation (6). A discrete version of the function involving 21 data points was saved in a lookup table. The pole at $\Delta f_{f_{a}}=0$ was arbitrarily replaced by a finite value of 100.

The measurement of a sinusoidal oscillation with the frequency $f_{a}$ over a limited period *T* corresponds to the multiplication of the sinusoid with a rectangular pulse, which is unity during the measurement period *T* and zero otherwise. The resulting Fourier spectrum $A_{s}(f)$ is the convolution of the spectra of the sinusoid and the rectangular pulse, which is a sinc-function located at $f_{a}$ and $-f_{a}$ (sinc x=sinx/x; Figure 5a). The FFT calculates the spectrum $A_{s}(f)$ at discrete frequencies, separated by $\Delta f_{S}=1/T$, thus producing a discrete spectrum $A_{\mathrm{sf}}(f)$. When the measurement time *T* is an integer multiple of the period $1/f_{a}$, the frequency sampling points coincide with the zero crossings of the sinc-function and its peak. In this case, $A_{\mathrm{sf}}(f)$ consists of a single spectral line at exactly the vibration frequency $f_{a}$.

Usually, the measurement time *T* is not an integer multiple of $1/f_{a}$—we want to measure $f_{a}$ because we do not know it and, therefore, cannot choose *T* as a multiple of $1/f_{a}$. In this situation, the discrete spectrum comprises many more than just one spectral line, a result of sampling the sinc-function (leakage effect, Figure 5a). The maximum $|A_{\mathrm{sf}}{|}_{\max}$ of the discrete amplitude spectrum does not occur at the frequency $f_{a}$, but is shifted by $\Delta f_{f_{a}}$ (picket fence effect [35] (pp. 1–23)). The oscillation frequency $f_{a}$ of a sinusoid, the spectrum of which is not distorted by nearby spectral components, can be extracted from the values of the discrete spectrum involving leakage effects. We propose a novel energy-efficient algorithm for this. It uses the fact that the two largest values of the discrete amplitude spectrum in the vicinity of a resonance peak are related by:

(6) $$\frac{|A_{\mathrm{sf}}{|}_{\max}}{|A_{\mathrm{sf}}{|}_{\max,2}}=\frac{\left|\sin c(\pi \cdot \Delta f_{f_{a}}/\Delta f_{S})\right|}{\left|\sin c(\pi (\pm 1+\Delta f_{f_{a}}/\Delta f_{S}))\right|}.$$

For a quick calculation of $\Delta f_{f_{a}}$, this function was evaluated at 21 points (Figure 5b) and the values were saved in a lookup table (LUT) in the MCU storage. This is used to determine the shift $\Delta f_{f_{a}}$ and thus an improved value of $f_{a}$ from the two largest components in the measured vibration spectrum.

**Figure 6:**
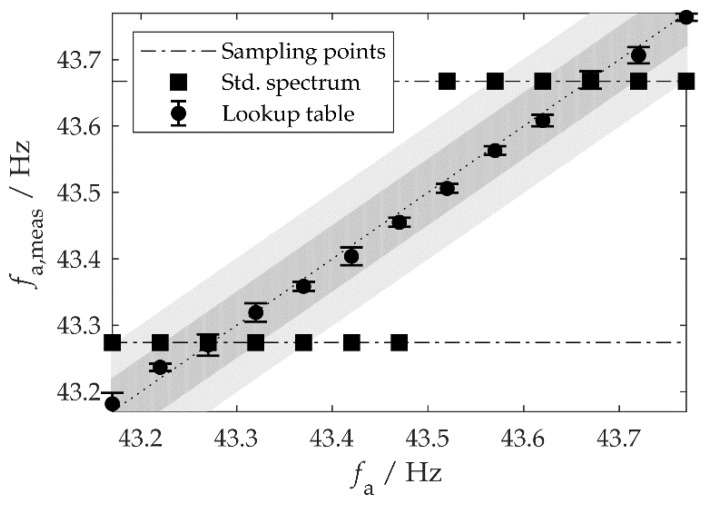
Vibration frequencies *f*_a,meas_ measured by our system when excited with different frequencies *f*_a_. Each point is the average of five individual measurements (95% confidence level). The dark and light gray bands represent respective tolerance bands of ±0.05 Hz and ±0.1 Hz around the true value (dotted line).

To test the algorithm, the acceleration sensor was excited on the test bench with a harmonic vibration (amplitude $A=1\;{m/s}^{2}$, $f_{a}\approx 43\;\mathrm{Hz}$). During the time T=2.56 s the sensor measured 1024 values. Figure 6 shows the frequencies $f_{a,\mathrm{meas}}$, which the MCU determined from the measured values, both for evaluation without post-processing ($\Delta f_{S}=0.4\;\mathrm{Hz}$) and for the new method using the LUT. Each frequency $f_{a}$ was measured five times. Without post-processing, the measured oscillation frequency was restricted to the discrete sampling frequencies, i.e., to integer multiples of $\Delta f_{S}$. The deviation from the true vibration frequency $f_{a}$ can be up to $\frac{1}{2}\Delta f_{S}=0.2\;\mathrm{Hz}$ and therefore violated the requirement. In contrast, the measured frequencies evaluated by the LUT method deviated by less than 0.05 Hz from the true values (including the measurement uncertainty), which met the requirement. Without post-processing, a measurement time of at least T<10 s would be necessary to achieve this accuracy. The MCU only needed 44 ms to receive and process the measurement data. The data are transmitted in blocks every 100 ms, with the MCU being in standby mode in between. 

This novel frequency determination works well for clearly separated harmonic vibrations, but not with broadband excitation. This perfectly matches the characteristics of our vibration sources (see Section 1) [12,13].

### 2.5. Loop Algorithm

The faster the system detects a change in the vibration frequency and adapts its resonance frequency accordingly, the less energy is lost during the harvest. This contrasts with the demand for the low energy consumption of the control mechanism. Depending on the characteristics of the environment in which the harvester is to be used, it is important to find a compromise. For instance, the determination of $f_{a}$ every 5 s ensures a quick response, but the energy consumption increases significantly. And a periodic adaptation is certainly inefficient when the vibration frequency changes on a timescale of the order of a second. In this work, a time interval of 30 s between two measurements was assumed, as this describes situations in which one may realistically hope to operate self-adaptive energy harvesters.

**Figure 7:**
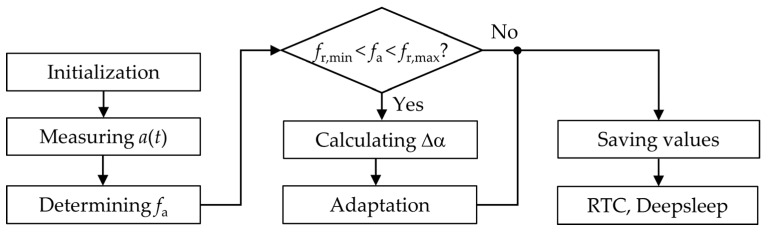
Algorithm for the automatic adaptation of the energy harvester. Real time clock (RTC).

Figure 7 shows the algorithm used for the automatic adaptation in our harvesting system. After a deep-sleep period, the MCU wakes up and runs some initializations. Then, the sensor measures the acceleration a(t) and transmits the data to the MCU, which then determines the dominant vibration frequency $f_{a}$ with the method presented in Section 2.4. This takes about T=2.56 s. If $f_{a}$ is inside the adaptation range $f_{r,M}\pm \Delta f_{\mathrm{AB}}/2$, the MCU calculates the required angle change Δα and the motor steps based on the actual and the required magnet angles. Subsequently, the MCU initializes the stepper motor driver and the adaptation is performed in less than 100 ms. Before setting the real time clock (RTC) for a wakeup after 30 s and going into deep sleep, important operation values required for the code are saved in the RTC backup register. 

In this setting, the average consumption of the complete system was measured to be 22 µW at a clock rate of 16 MHz and with a 3 V power supply. The standby power consumption of 1–2 µW is included in this value.

## 3. Results

### 3.1. Experimental Setup

The self-adaptive energy harvester was characterized on the test bench described in Section 2.2. The test bench simulated a time-varying vibration source with a sequence of six frequencies $f_{a,1}$ to $f_{a,6}$. The time interval between two frequency changes (duration of stationarity) was τ. After the end of a sequence, $f_{a,6}$ is followed by $f_{a,1}$ and the sequence restarts. The average adaptation width ${\bar{\Delta f}}_{r}$ results from the spacing between the frequencies. With uniformly distributed random frequencies within the adaptation range, one would have ${\bar{\Delta f}}_{r}=\Delta f_{\mathrm{AB}}/3$ [31]. Furthermore, in the case of a periodic adaptation obeying Equation (4), the average angle shift is $\bar{\Delta \alpha }=45{}^{\circ}$ (see Appendix A). The sequence $f_{a,1}=36.4\;\mathrm{Hz}$, $f_{a,2}=33.0\;\mathrm{Hz}$, $f_{a,3}=34.4\;\mathrm{Hz}$, $f_{a,4}=40.1\;\mathrm{Hz}$, $f_{a,5}=38.5\;\mathrm{Hz}$ and $f_{a,6}=35.1\;\mathrm{Hz}$ fulfills the two conditions ${\bar{\Delta f}}_{r}=\Delta f_{\mathrm{AB}}/3$ and $\bar{\Delta \alpha }=45{}^{\circ}$ and thus approximates a random, uniformly distributed sequence. The acceleration amplitude *A* was kept constant during each experiment. The energy content $E_{C}(t)$ of the capacitor was calculated from the capacitor voltage: $E_{C}(t)=\frac{1}{2}CU_{C}^{2}(t)$. The capacitor was pre-charged to $U_{C}(t=0)=2.5\;V$. The harvester was tested with τ = 2, 5 and 10 min and *A* = 0.8, 1.0 and 1.2 ${m/s}^{2}$. Every experiment started with the center frequency $f_{a,1}=f_{r,M}=36.4\;\mathrm{Hz}$, to which the harvester was perfectly adjusted. After the time τ, the vibration frequency $f_{a}$ was changed according to the above sequence. The MCU determined the vibration frequency every 30 s and carried out the adaptation when $f_{a}$ had changed. All parts of the system, including the stepper motor, are powered by the harvested energy.

### 3.2. Periodic Adaptation

**Figure 8:**
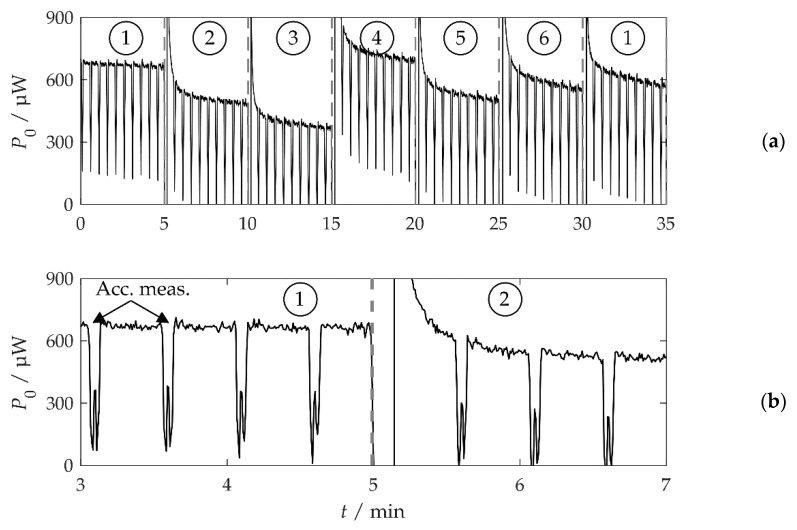
Harvested power *P*_0_ of a sequence with *A* = 1 m/s² and τ = 5 min. The numbers in circles mark the time intervals *i* during which the vibration frequency was constant at *f*_a,*i*_. The interval boundaries are marked by gray dashed lines. (**a**) The complete sequence. (**b**) Detail of (**a**) in the vicinity of the first adaptation step.

Figure 8a shows the harvested power $P_{0}$ for $A=1\;{m/s}^{2}$ and τ = 5 min for a complete sequence. The power was calculated from the storage voltage $U_{C}$. $P_{0}$ was constant at around 650 μW in time interval 1, and was only periodically disturbed by the acceleration measurement every 30 s. Figure 8b shows $P_{0}$ in the vicinity of the first adaptation step after the ambient vibration frequency changed from $f_{a,1}=36.4\;\mathrm{Hz}$ to $f_{a,2}=33.0\;\mathrm{Hz}$ at *t* = 5 min. After the change, the harvested power decreased to a negative value because the resonance frequency $f_{r}=36.4\;\mathrm{Hz}$ no longer matched the excitation and because of the a slight leakage current of the storage. After the subsequent acceleration measurement, the system recognized the change in $f_{a}$ and adapted its resonance frequency to $f_{r}=f_{a,2}$ with the rotation of the tuning magnet. The harvested power again rose to $P_{0}=550\;\mu W$. The high power $P_{0}$ immediately after the adaptation was likely caused by the energy stored in the coil windings of the motor, which flew back into the storage after the motor rotation. $f_{r}$ did not perfectly match $f_{a}$ at every time interval, so $P_{0}$ ranged between 450 and 700 μW (Figure 8a). The decrease in $P_{0}$ towards the end of some time intervals was due to the leakage current, which increased with the storage voltage $U_{C}$ due to the harvested energy.

**Figure 9:**
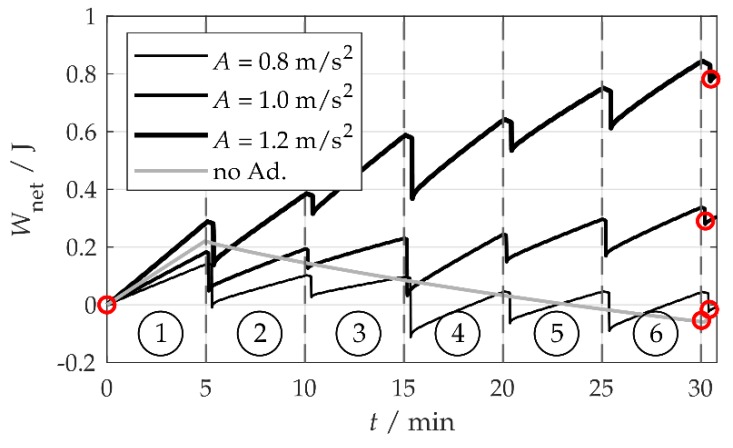
Net harvested energy *W*_net_ at a stationarity duration of τ = 5 min for different acceleration amplitudes *A*. The red circles mark calculation points for the net power *P*_net_.

The net harvested energy $W_{\mathrm{net}}=W_{0}-W_{T}$ is shown for different amplitudes *A* in Figure 9. $W_{\mathrm{net}}$ was calculated from the capacitor energy $E_{C}$ according to $W_{\mathrm{net}}(t)=E_{C}(t)-E_{C}(t=0)$. Higher acceleration amplitudes *A* increased the energy harvest $W_{0}$ during a full sequence, owing to the higher harvested power. Since the adaptation energy $W_{T}$ did not depend on *A* and was identical for all the curves shown, the net harvested energy $W_{\mathrm{net}}$ also increased with *A*. $W_{\mathrm{net}}$ and $P_{\mathrm{net}}$ were read out by comparing the energy value $W_{\mathrm{net}}$ at the start of the first time interval ($f_{a,1}$) of the first sequence run (which, here, is always $W_{\mathrm{net}}=0$) to the starting value of the first time interval of the second sequence run. This is illustrated by red circles in Figure 9.

With acceleration amplitudes of $A=1.0\;{m/s}^{2}$ and $1.2\;{m/s}^{2}$ and τ = 5 min, the net harvested energy was positive, whereas for $A=0.8\;{m/s}^{2}$, the adaptation was inefficient. Without optimizing the adaptation settings, this system would have had to deactivate the adaptation. At $A=1.0\;{m/s}^{2}$, the harvester set to $f_{r}=36.4\;\mathrm{Hz}$ with deactivated adaptation was tested for comparison. The harvester was able to harvest energy in the first time interval ($f_{a,1}=f_{r}=36.4\;\mathrm{Hz}$), but not in the other intervals, during which the vibration frequencies did not match the resonance frequency of the harvester. At the first time interval, the harvested power of the non-adapting system was slightly higher than that of the adapting system, as the non-adapting system saved the power needed to periodically measure the vibration frequency. At the other time intervals, the net energy $W_{\mathrm{net}}$ decreased as a consequence of the leakage current. Leakage was present in all tests, so a comparison of the net power $P_{\mathrm{net}}$ based on the sequence time is permissible. The specific results are listed in Table 1. The non-adaptive system harvested $P_{\mathrm{net}}=-33\mu W$ at $A=1.0\;{m/s}^{2}$.

At $A=1.0\;{m/s}^{2}$ and τ = 5 min, the self-adapting system harvested 300 mJ more energy per sequence than the non-adapting system and was therefore superior to it.

**Figure 10:**
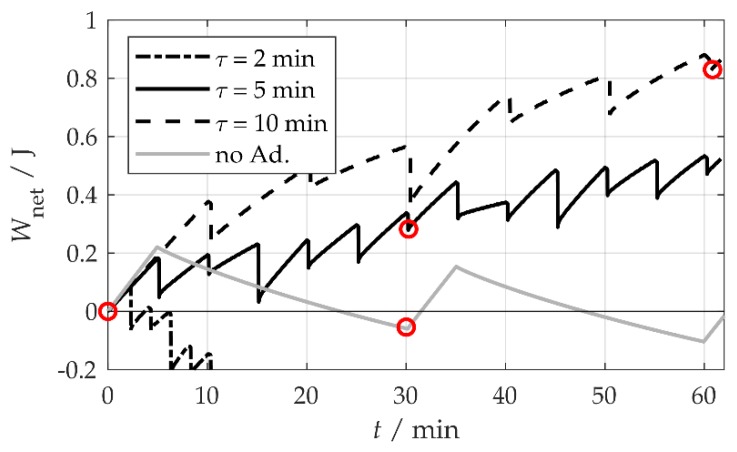
Net energy *W*_net_ at *A* = 1.0 m/s² for different stationarity durations τ. The red circles mark calculation points for the net power *P*_net_. The sequence duration was *t* = 6τ.

The tests were also carried out for other stationarity durations τ. Figure 10 shows the net energy harvest at $A=1.0\;{m/s}^{2}$ for τ = 2, 5 and 10 min and, for comparison’s sake, for τ = 5 min without adaptation. At τ = 10 min, the sequence duration was 60 min, so two sequences for τ = 5 min were recorded for comparison. Thus, with a similar harvested power $P_{0}$ in all experiments, it can be seen that, in more dynamic environments with a smaller τ, the energy cost for regular adaptation increases and, consequently, $W_{\mathrm{net}}$ and $P_{\mathrm{net}}$ decrease. The specific results can be found in Table 1.

At $A=0.8\;{m/s}^{2}$ and τ = 2 min, the voltage $U_{C}$ dropped below the threshold voltage $U_{C,\min}=1.9\;V$ (below $U_{C,\min}$, the MCU is not supplied anymore) before the end of the sequence. At $A=1.2\;{m/s}^{2}$ and τ = 10 min, $U_{C}$ reached the upper limit $U_{C,\max}=3.8\;V$ before the end of the sequence. In both cases, it was therefore not possible to provide reliable averages.

### 3.3. Interpretation

As corroborated by the data in Table 1, a more dynamic environment, i.e., an environment with more frequent frequency changes characterized by shorter stationarity durations τ, requires more frequent adaptations. Less net power $P_{\mathrm{net}}$ can be extracted from such an environment. The authors have already dealt with this issue theoretically in [31] and derived the equation:

(7) $$P_{\mathrm{net}}(\tau )=P_{0}-W_{T}/\tau .$$

where $W_{T}$ is the average energy value per adaptation. 

**Figure 11:**
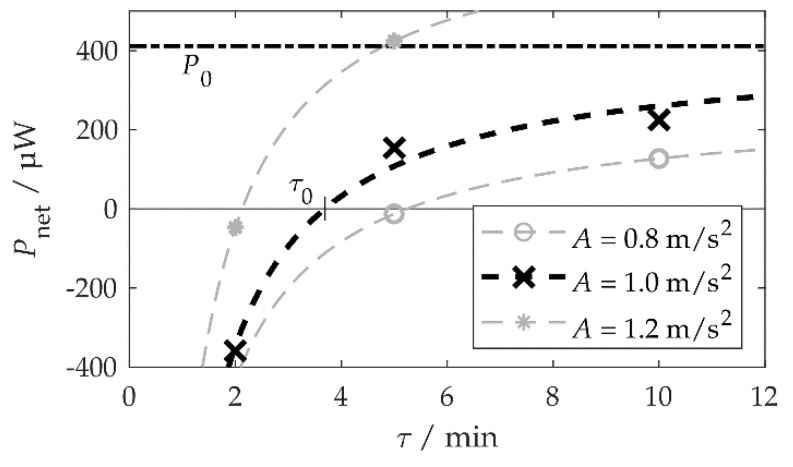
Net power *P*_net_ depending on the stationarity duration (length of the time interval between two subsequent ambient vibration frequency changes) τ. The dots mark the values from Table 1, the dashed lines are fit functions according to Equation (7). The value of the harvested power *P*_0_ for *A* = 1.0 m/s² is the fit function result. It can also be interpreted as the limit value of *P*_net_ for τ → ∞.

Figure 11 visualizes the measured values from Table 1 together with fit functions according to Equation (7) for each acceleration amplitude. At an amplitude of $A=1.0\;{m/s}^{2}$, the fit yielded $P_{0}=410\;\mu W$ and $W_{T}=91\;\mathrm{mJ}$. At $\tau_{0}=3.7\;\min$, the harvested and adaptation power would have been equal. The fit in Figure 11 corresponds to Figure 1 in [31].

According to Figure 8a, $P_{0}\approx 500–600$ μW would be expected. The lower value of 410 μW is attributed to several circumstances. Firstly, with more energy harvested, the storage voltage $U_{C}$ increased during the tests and so did the leakage current. Secondly, there was a time interval between the change in the vibration frequency $f_{a}$ and the adaptation of the system, during which no harvest was possible (Figure 8b). The average adaptation energy $W_{T}=91\;\mathrm{mJ}$ was slightly higher than the 76 mJ calculated according to Equation (5), with Δα=45°.

In summary, the self-adaptive system, in most cases, harvested more energy than the non-adaptive system. Only in highly dynamic environments it is preferable to switch off the adaptation.

### 3.4. Generalization

This work presents the results of a specific system in which general characteristics and individual strengths and weaknesses blur. This section separates general and concrete properties.

A self-adaptive kinetic energy harvester works best when the harvested power $P_{0}$ for a particular excitation is higher and the energy $W_{T}$ required for adaptation is smaller, so that the available net power $P_{\mathrm{net}}$ according to Equation (7) is as high as possible. The combination of $P_{0}$, $W_{T}$ and the stationarity duration τ determines if the operation of the system is meaningful.

The harvested power $P_{0}$ is a result of the design of the harvester, i.e., its geometry and electromechanical coupling. In general, a harvester with a larger seismic mass *m* would produce a higher harvested power. $P_{0}$ decreases in the presence of leakage currents and when power is required to measure the vibration frequency. However, the latter effect amounted to only 22 μW in the demonstrator system.

The adaptation was realized by a stepper motor, which is large in comparison to the harvester dimensions, but despite its size it consumed very little energy, meaning that an adaptation was possible at intervals of a few minutes. The choice of a smaller motor causes a smaller residual torque, at least with a comparable motor design. As a result, the axial forces $F_{A}$ have to be reduced, which leads to a reduction in the adaptation bandwidth $\Delta f_{\mathrm{AB}}$. In principle, of course, other adaptation methods could be used [11,18].

Let us also mention that our demonstrator system did not measure the angular position of the motor shaft or the tuning magnet. The knowledge of the angular position would be advantageous in order to detect step losses and to make the system functional again after a failure, such as a drop in the storage voltage to below a minimum value. One would have to check if the extra energy needed to operate such an angle measurement system was admissible.

## 4. Summary and Outlook

This work presents a kinetic self-adaptive energy harvesting system that works completely self-sufficiently, i.e., without user intervention and without external energy. The system harvests vibration energy with electromagnetic coupling. The resonance frequency of the harvester can be changed by a diametrically magnetized tuning magnet that changes the axial mechanical load on a bending beam structure depending on the angle of rotation. This adaptation mechanism is inspired by the work of Hoffmann et al. [32]. The harvester adapts its resonance frequency between 32.2 and 40.6 Hz, which is equivalent to a relative tuning range of 23%.

The system is the first self-adaptive energy harvester described in the literature that works completely self-sufficiently with passive adaptation (without energy needed to maintain a constant resonance frequency). This harvester does not have to interact with an external intervention for operation, so it could supply power to an electrical load, such as a WSN node. Among other things, this required the installation of an energy-efficient and fast-frequency measurement mechanism. With a measurement time of 2.5 s, the system can continuously determine the dominant vibration frequency with a deviation of less than 50 mHz and a power consumption of 22 µW.

Theoretical considerations about the influence of the dynamics of frequency changes on the usable harvested power [31] could be experimentally corroborated with this setup. The self-adaptive system was tested with different acceleration amplitudes between 0.8 and $1.2\;{m/s}^{2}$ and time durations between two frequency changes from 2 to 10 min. The system was superior to a non-adaptive system when the frequency changes occurred every 5 min or less frequently. 

Our system was successfully examined on a vibrational test bench. In a future work, we plan to test the installation of the harvester on a real vibration source. This involves the adaptation of the harvester geometry to include the vibration frequencies in the tuning range and the optimization of the operation parameters such as the length of the measurement cycle.

## Figures and Tables

**Table 1:** Average net power *P*_net_ of a sequence for different acceleration amplitudes *A* and stationarity durations τ.

***P*_net_/µW**	**τ = 2 min**	**τ = 5 min**	**τ = 10 min**
***A* = 0.8 m/s²**	— ^1^	−13	127
***A* = 1.0 m/s²**	−359	154	225
***A* = 1.2 m/s²**	−47	424	— ^1^

^1^ No reliable average values.

## Appendix A

According to [31], the average frequency spacing ${\bar{\Delta f}}_{r}$ between the resonance frequency of a harvester before and after an adaptation step ($f_{r}$ and $f_{a}$) is ${\bar{\Delta f}}_{r}=\Delta f_{\mathrm{AB}}/3$, where $\Delta f_{\mathrm{AB}}$ denotes the adaptation bandwidth. It is assumed that the frequencies are random variables and are uniformly distributed in the adaptation range.

According to Equation (4), there is a trigonometric relationship $f_{r}(\alpha )$ between the resonance frequency $f_{r}$ and the angle of rotation α of the tuning magnet (0°≤α≤180°). If the frequency range is normalized, such that the lower and upper limit frequency of the adaptation range respectively correspond to the values zero and one, the normalized frequency *y* is described by:

(A1) $$y(\alpha )=\frac{1}{2}\cdot (1-\cos\alpha ).$$

The function $y(\alpha_{G})$ also represents the cumulative probability distribution function $P(0{}^{\circ}\leq \alpha \leq \alpha_{G})$ where $0{}^{\circ}\leq \alpha_{G}\leq 180{}^{\circ}$. $\alpha_{G}$ is the upper limit of the probability interval. Thus, the probability density function is:

(A2) $$p(\alpha_{G})=\frac{dP(\alpha_{G})}{d\alpha_{G}}=\frac{1}{2}\cdot \sin(\alpha_{G}).$$

The average distance between the angles $\alpha_{a}$ and $\alpha_{r}$, which correspond to the uniformly distributed frequencies $f_{a}$ and $f_{r}$, is:

(A3) $$\begin{array}{l} \bar{\Delta \alpha }=E(|\alpha_{a}-\alpha_{r}|)=\int_{f_{r}=0{}^{\circ}}^{180{}^{\circ}}\int_{f_{a}=0{}^{\circ}}^{180{}^{\circ}}p(\alpha_{a})\cdot p(\alpha_{r})|\alpha_{a}-\alpha_{r}|df_{a}\;df_{r}=\\ \;=\frac{1}{4}\int_{f_{r}=0{}^{\circ}}^{180{}^{\circ}}\int_{f_{a}=0{}^{\circ}}^{180{}^{\circ}}\sin(\alpha_{a})\cdot \sin(\alpha_{r})\cdot \;|\alpha_{a}-\alpha_{r}|df_{a}\;df_{r}=45{}^{\circ}. \end{array}$$
